# Evidence of Evolutionary Constraints That Influences the Sequence Composition and Diversity of Mitochondrial Matrix Targeting Signals

**DOI:** 10.1371/journal.pone.0067938

**Published:** 2013-06-25

**Authors:** Stephen R. Doyle, Naga R. P. Kasinadhuni, Chee Kai Chan, Warwick N. Grant

**Affiliations:** La Trobe Institute for Molecular Sciences, La Trobe University, Bundoora, Australia; University College Dublin, Ireland

## Abstract

Mitochondrial targeting signals (MTSs) are responsible for trafficking nuclear encoded proteins to their final destination within mitochondria. These sequences are diverse, sharing little amino acid homology and vary significantly in length, and although the formation of a positively-charged amphiphilic alpha helix within the MTS is considered to be necessary and sufficient to mediate import, such a feature does not explain their diversity, nor how such diversity influences target sequence function, nor how such dissimilar signals interact with a single, evolutionarily conserved import mechanism. An *in silico* analysis of 296 N-terminal, matrix destined MTSs from *Homo sapiens*, *Mus musculus, Saccharomyces cerevisiae, Arabidopsis thaliana*, and *Oryza sativa* was undertaken to investigate relationships between MTSs, and/or, relationships between an individual targeting signal sequence and the protein that it imports. We present evidence that suggests MTS diversity is influenced in part by physiochemical and N-terminal characteristics of their mature sequences, and that some of these correlated characteristics are evolutionarily maintained across a number of taxa. Importantly, some of these associations begin to explain the variation in MTS length and composition.

## Introduction

It is generally accepted that mitochondria have evolved from an alpha-proteobacterium that was engulfed by an ancestral eukaryotic cell over one billion years ago. Over time, almost all of the original bacterial genes have been translocated, such that now the majority of mitochondrial proteins are transcribed in the nucleus, translated in the cytoplasm and are actively trafficked, potentially through multiple cellular compartments, to reach their final destination. The gradual loss of autonomy to the nucleus would have required a number of independent events to occur [1], [2]: the transfer and integration of bacterial-derived genetic information into the nuclear genome, followed by genetic modifications to allow nuclear transcription and regulation, translation on cytoplasmic ribosomes and lastly, trafficking of the protein to its correct destination within the mitochondria. This trafficking process is mediated by multiple molecular interactions between the import apparatus and a mitochondrial targeting signal (MTS) sequence, a ‘molecular address’ that facilitates import and sorting to its correct destination (see Chacinska et al., [3], Mokranjac and Neupert [4] and Schleiff and Becker [5] for comprehensive descriptions of the import pathway).

Although it is nearly a quarter of a century since they were initially described [6]–[8], MTSs remain poorly characterised. MTSs from different proteins share virtually no sequence homology and vary extensively in length; proteins targeted to the mitochondrial matrix do however typically contain an N-terminal MTS and that these targeting sequences are loosely related by some characteristic physicochemical properties, including being rich in hydroxylated and basic residues, deficient in acidic resides [9], and most likely exhibiting a tendency to form an amphiphilic alpha helix (i.e. with opposing positively charged and hydrophobic faces) [6]. Some simple residue conservation sometimes applies, such as the proposed φχχφφ motif (where φ is hydrophobic and χ is any other residue) thought to mediate recognition of the presequence by Tom20 [10], [11], and the -10/-3/-2 arginine motif [12], which has been proposed to be involved in cleavage site recognition by the mitochondrial processing peptidase (MPP). However, the determinants of cleavage recognition have not been fully elucidated as MTSs may contain either one or two cleavage sites (a second site recognised by the mitochondrial intermediate peptidase, MIP) or no cleavage site at all (i.e. the ‘mature’ protein retains the MTS in the matrix). These limited characteristics have been used in the development of software to predict signal sequences [13]–[16], however, the significant variation in both sequence composition and length of MTSs renders this task difficult, so that a predicted MTS must be verified experimentally. Furthermore, the majority of research dedicated to matrix protein import is focused primarily on understanding the molecular interactions between the components of the import translocases and the MTS is often overlooked, possibly due to the lack of obvious explanation for its diversity.

It is interesting to speculate on the nature of the selective pressures that may have been exerted on the development of any given matrix-targeted MTS. Matrix-destined MTSs have a functional role in multiple cellular compartments (cytoplasm, inner-membrane space and matrix space) as they interact with cytosolic chaperones and several different components of the membrane-bound mitochondrial import and processing machinery. Moreover, they direct a vast range of different proteins through a single import pathway to the matrix space. Although formation of an amphiphilic alpha helix within an N-terminal MTS is broadly described to be necessary and sufficient to direct import [4], [17], [18], it seems to be an overly simple explanation for a complex role, and is unlikely to be relevant in all stages of import [19]. Given the significant variation among MTSs and the complex processes they facilitate, it is more likely that there are additional functionally significant MTS characteristics that have not been described.

In this investigation, an *in silico* analysis of N-terminal, matrix-destined MTSs from five diverse taxa (*Homo sapiens*, *Mus musculus, Saccharomyces cerevisiae, Arabidopsis thaliana*, and *Oryza sativa*) was undertaken to search for evolutionarily conserved characteristics that could shed light on the evolutionary relationships between diverse MTSs, and/or, between an individual MTS and the protein that it imports. We hypothesised that sequence characteristics of the mature portion of the protein to be imported are responsible for the extensive variation in MTS sequence composition and length. A number of physicochemical correlations were identified that support this hypothesis, and these results are discussed in reference to the evolutionary adaptation required of MTSs to enable efficient mitochondrial import.

## Methods

### Data mining

A sequence database of mitochondrial matrix-localised proteins was constructed in November 2012 from sequences retrieved from NCBI GenBank using Gene Ontology with the keywords ‘mitochondrial matrix’ and UniProt (http://www.uniprot.org/) databases, as well as from other sources including Appendix 4 of Methods in Cell Biology, Vol. 65, and websites such as the Human Mitochondrial Protein Database (http://bioinfo.nist.gov/hmpd/index.html) and MitoProteome Database (http://www.mitoproteome.org/). A large proportion of the *S. cerevisiae* [20], and all of the *A. thaliana* and *O. sativa* [21] sequences were obtained from previously published proteomics databases. Each candidate sequence was required to meet the following criteria: (i) the mature protein is imported and localised within the mitochondrial matrix, (ii) the mature protein is not part of the inner-mitochondrial membrane (iii), the sequence has a defined N-terminal targeting signal, and (iv) the sequence was catalogued within the NCBI protein reference sequence database, which was used to acquire accession numbers and to extract the amino acid sequence. Dataset S1 contains a complete list of the mitochondrial matrix proteins, gene IDs, accession numbers, MTS sequences and their mature primary amino acid sequences for all sequences used in this study.

### Primary amino acid sequence analysis

Full length sequences were broken into two sub-sequences; the first containing the MTS and the second containing the remaining portion of the sequence found in the mature protein (designated ‘mature sequence’ or ‘mtpt’ throughout this study). Amino acid composition, length, and physicochemical characteristics were determined using a Perl program, *AAResidueFreqCalculator*, which enabled frequencies to be determined from a FASTA sequence input (see File S1 for Perl script and running details). Charge characteristics at pH 7.5 were calculated using CLC Genomics Workbench (5.5.1). Isoelectric point (pI) was calculated using the Compute pI/Mw tool (http://web.expasy.org/cgi-bin/compute_pi/pi_tool). Secondary structure analysis was undertaken using the tools PROFsec and MD (www.predictprotein.org, [22]).

A reduced amino acid alphabet [23] was developed to analyse physicochemical characteristics in their primary amino acid sequence context, which enabled the investigation of amino acid residue-type effects (as opposed to individual amino acid residue-specific effects). The 20-letter amino acid code was divided into nine groups; non-polar aliphatic (G, A, V, I, L), non-polar aromatic (F, W), non-polar cyclic (P), polar sulphur containing (C, M), polar hydroxyl (S, T), polar aromatic (Y), polar acidic-amide (N, Q), acidic (D, E), and basic (R, H, K), according to the biochemical properties explorer at NCBI (http://www.ncbi.nlm.nih.gov/Class/Structure/aa/aa_explorer.cgi). Conversion of each amino acid sequence from the standard alphabet to the reduced amino acid alphabet was performed using the Perl script, *ReducedAASequenceConverter* (see File S2 for Perl script and running details) prior to further analysis.

### Statistical analyses

Data analysis, including multivariate principal component analysis (PCA), Spearman's rho correlation and regression analysis, Kruskal-Wallis test, and graph construction was performed using SPSS (version 17). All other data manipulation and analysis was performed in Microsoft Excel (2010).

## Results and Discussion

### Multivariate analysis of MTSs and associations with their mature sequence

A database and literature search yielded an extensive list of mitochondrial matrix proteins, of which a total of 296 candidates from five diverse taxa met the defined selection criteria. This list was comprised of two mammalian species, *H. sapiens* (85 sequences) and *M. musculus* (84), the bakers yeast *S. cerevisiae* (56), and two plant species, the monocot *O. sativa* (36) and the dicot *A. thaliana* (35). The primary amino acid sequence for each protein was divided into two sequences (MTS and the mature sequence), after which a range of sequence properties was determined for each MTS/mature protein pair. These properties included sequence length and charge (pI and charge at pH 7.5), as well as the proportion of nine groups of amino acids segregated based on shared physicochemical characteristics that may influence potential functional properties of the sequence (Table S1).

**Figure 1:**
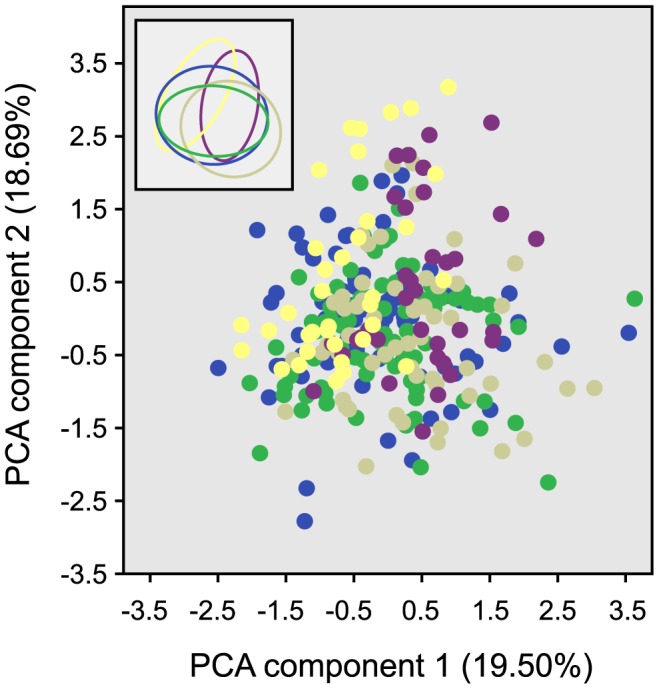
Comparison of MTS diversity among five taxa using multivariate PCA. The first two components are plotted, which represent 38.19% of variation among MTS sequences. Insert depicts simplified version of the scatterplot to emphasise boundaries of distribution. Blue – *H. sapiens*, Green – *M. musculus*, Beige – *S. cerevisiae*, Purple – *A. thaliana*, Yellow *– O. sativa*.

To analyse these characteristics, multivariate principle component analysis (PCA) was used to explore variation among the five species. Five components were found to explain 71.47% of the variance among MTS; sequence length and charge at pH 7.5 (19.5%), non-polar aliphatic and aromatic residues (18.69%), polar aromatic and acidic-amide residues (12.51%), polar hydroxyl and basic residues (11.51%), and finally acidic residues (9.61%). A pairwise comparison of the first two components demonstrated that all five species had diverse but somewhat overlapping distributions that reflect their phylogenetic history, with *H. sapiens* and *M. musculus* most similar, followed by *S. cerevisiae* and then both plant species (Figure 1). Some subtle differences in variance were evident, particularly between *O. sativa* and *A. thaliana*, which shared only a small proportion of their distribution. These subtle differences between the two plant species remained consistent across all pairwise PCA comparisons (Figure S1). Differences in MTS composition, particularly surrounding the cleavage site [21], and in Tom20 genomic copy number (*A. thaliana* contains four copies of the Tom20 gene whereas *O. sativa* only contains a single copy [24], [25]) between both plant species have been described previously.

To investigate the hypothesis that features of a mature sequence influence the composition of the associated MTS, both MTS and mature sequence characteristics were analysed together using PCA. A significant positive association between both sequence length and charge at pH 7.5 of MTSs and the proportion of acidic residues in the mature sequence was found in the *H. sapiens*, *M. musculus* and *S. cerevisiae* datasets. A similar positive correlation between MTS length and charge and the mature sequence acidic residue content was seen in the *O. sativa* dataset, however, the charge association was due to a correlation with MTS basic residues rather than charge at pH 7.5 as seen in the *H. sapiens*, *M. musculus* and *S. cerevisiae* datasets. Acidic residues of the import machinery have been shown to be essential for import [26]–[31] and offer potential interaction sites for a positively charged MTS. These essential acidic regions, and the cation-selective nature of Tom40 [32], are likely responsible for the characteristic deficiency of acidic residues within the MTS due to negative selection from electrostatic repulsion, as import would be inhibited if the MTS also contained a significant proportion of acidic residues. The residue composition of the mature sequences did not significantly differ from the global average amino acid composition of the proteome (data not shown), and therefore, mature sequences that contain and require acidic residues for normal structure and function would experience some electrostatic repulsion as they proceed though the import complexes following passage of the MTS. It is therefore possible that a longer MTS associated with more acidic mature sequence would traverse the outer membrane (via Tom40) and engage the membrane potential and mtHsp70 [33] at the inner membrane earlier relative to a shorter MTS, and thereby may permit the import machinery to overcome the potentially inhibitory interaction between acidic residues of mature proteins and the import complexes. Interestingly, the proportion of acidic residues found in the mature sequence did not correlate with any property of MTSs within the *A. thaliana* dataset. This may relate to the amino acid composition of the *A. thaliana* import complexes: TOM components from *A. thaliana* contain fewer acidic residues compared to the more acidic TOM components of yeast [34], and therefore *A*. *thaliana* mature sequences would not be inhibited by negative charge repulsion to the same extent as yeast mature sequences. The most prominent correlation within the *A. thaliana* group of sequences suggests that MTS length is positively associated with mature sequence length, a correlation that was not present in any of the other species (apart from a minor association in yeast). There was a smaller negative association between basic residues and pH 7.5 in the MTS and the charge at pH 7.5 in the mature sequence, which suggests that associations involving sequence charge may be present but are likely different than that observed in the other species.

### Pairwise correlation analysis of MTS and its mature sequence

**Figure 2:**
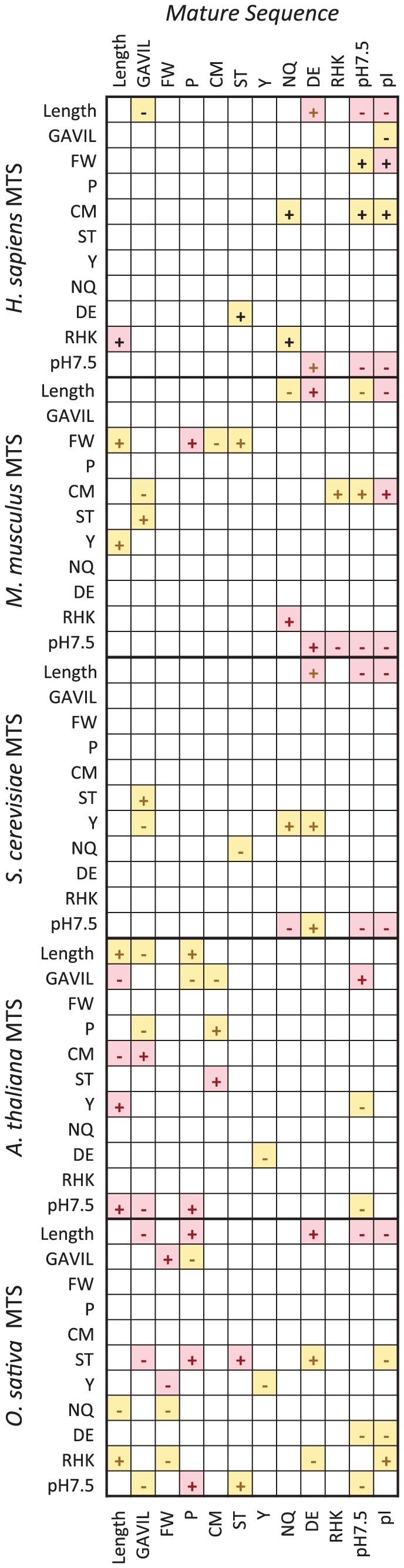
Correlation matrix to analyse pairwise characteristics between MTS and mature sequence. Spearman's rho correlation coefficients were calculated for each pair of variables between MTS (y-axis) and mature (x-axis) sequences for each species. Positive (+) and negative (-) correlations are indicated within each square. Yellow shading indicates p<0.05, red shading indicates p<0.01. Blank squares indicate no significant correlation was present between the pair of variables.

**Figure 3:**
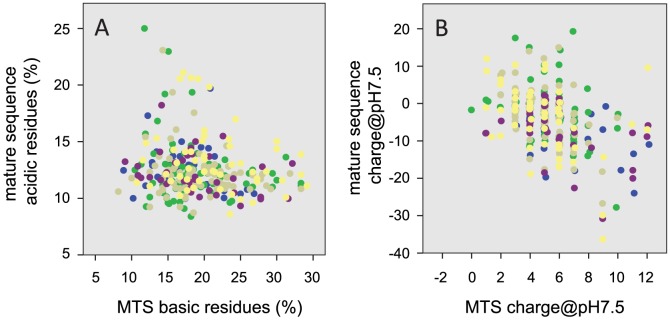
Correlation analysis of charge characteristics between MTS and mature sequences. Scatterplots were generated to investigate (A) basic residue composition (percentage of total) of the MTS against acidic residue composition of the mature sequence, and (B) global sequence charge characteristics by comparing charge at pH 7.5 of both MTS and mature sequences. Blue – *H. sapiens*, Green – *M. musculus*, Beige – *S. cerevisiae*, Purple – *A. thaliana*, Yellow *– O. sativa*.

To investigate these observations further and to determine the significance of each association, a pairwise Spearman's rho correlation matrix was generated for each species to directly compare properties of both MTS and mature sequences (Figure 2). Similar patterns of association to the multivariate analysis were observed; MTS length was significantly correlated with charge characteristics of the mature sequence (negative correlations with mature sequence pH 7.5 and pI), and particularly acidic residue content in all species but *A. thaliana*. It has been speculated previously that charge characteristics influence mitochondrial import rate, whereby a mitochondrial isoform (pI 9–9.5) of aspartate aminotransferase was imported up to four times faster than its cytosolic isoform (pI 6.7) when fused to an identical MTS sequence [35], [36]. Only a single correlation was shared among all five species; MTS charge at pH 7.5 was negatively correlated with the mature sequence charge at pH 7.5 (r_s_ range: -0.484>−0.372), which suggests that mature sequences that are more negatively charged tend to be associated with MTSs that are more positively charged. Basic residues have long been acknowledged to play an important role within the MTS, as they are thought to enable electrophoretic mobility of the precursor through the inner-membrane space in response to the membrane potential [37], which in turn increases the likelihood of MTS interaction with the import motor by initiating translocation through the Tim23 complex [38]. It is therefore surprising that there was little evidence from this analysis that positively charged basic content in the MTS was associated with the proportion of negatively charged acidic residues in the mature sequence (r_s_ = −0.108, p = 0.062; Figure 3A), leading us to conclude that the significant negative correlation between both MTS and mature sequence charge at pH 7.5 (r_s_ = −0.437, p<0.001; Figure 3B) is likely indicative of the balance between positive- and negatively charged residues in each sequence rather than being significantly influenced by any one individual amino acid property (basic or acidic) alone.

The pairwise analysis revealed some correlations that were unique to plants, and so emphasised further the difference between *A. thaliana* and *H. sapiens*, *M. musculus* and *S. cerevisiae* seen in the multivariate analysis. Both plant species show positive associations between MTS length and mature sequence non-polar cyclic residues (P), and in addition, MTS pH 7.5 and mature sequence non-polar aliphatic (GAVIL, negative correlation) and cyclic residues (P), all of which are not correlated in the non-plant species. A number of differences have been previously described that differentiate plant and non-plant species, and considerable differences between *Arabidopsis* and yeast MTS length and composition [39] are consistent with the results presented here. Moreover, phylogenetic analysis of mitochondrial import components suggests that although they are functionally similar, plant and animal Tom20′ are evolutionarily distinct, having undergone convergent evolution following the divergence of the animal and plant lineages [40], [41]. Some differences between plant and non-plant import mechanisms are likely due to the need to maintain specificity during the co-evolution of chloroplast and mitochondrial targeting in plant species [21]. However, the observation that *O. sativia* shares a number of correlated characteristics with both *A. thaliana* and non-plant species that were not otherwise shared (between *A. thaliana* and non-plant species; Figure 2) may be evidence that additional convergent evolutionary processes may have occurred in the development of the trafficking pathways between species.

### Influence of the mature sequence N-terminal composition on MTS diversity

**Figure 4:**
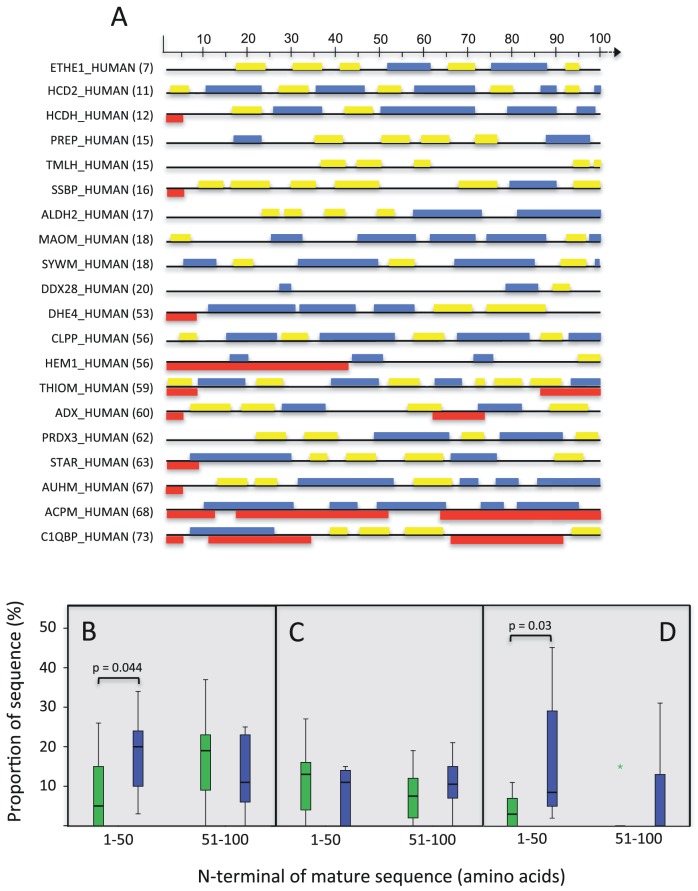
N-terminal secondary structure analysis of *H. sapiens* mature sequences associated with the ten shortest and ten longest MTSs. (A) Individual sequence IDs are indicated with MTS length in parentheses. Scale bar represents amino acid residue position after the MTS cleavage site. Yellow boxes – alpha helix, Blue boxes – beta sheet, Red boxes – predicted disordered sequence. Figure was adapted from secondary structure predictions of indicated mature sequences generated at www.predictprotein.org. Quantitative analysis of the proportion of predicted alpha helix (B), beta strand (C), and disordered (D) structures in the N-terminal mature sequences of short (green box plots) and long (blue box plots) MTSs for the first and second 50 amino acids of the mature sequence. The distribution of each structural group was analysed using a Kruskal-Wallis test for independent samples.

There have been a number of carefully controlled studies that suggest that the N-terminal of the mature sequence can influence the rate of precursor protein import [42]–[45]. The data presented in this investigation demonstrate that significant associations exist between MTS and the corresponding mature sequence. However, as the data presented here are derived from average composition values across the whole protein, the significance of the correlations described here is likely to be underestimated if local sequence features rather than average global sequence composition in the mature sequence plays a role in influencing MTS diversity. Therefore, we investigated whether two of the correlated properties described above were maintained between the immediate N-terminal 40 amino acids of the mature sequence and the second 40 amino acids (41–80 amino acids after the MTS)(Table 1). All species except for *A. thaliana* showed a significant correlation between MTS length and acidic residue content in the first 40 amino acids of the mature sequence that is then lost in the second 40 amino acids. *A. thaliana* shows an opposite effect; there is no association between MTS length and acidic residues in the first 40 amino acids, however, a significant correlation is evident in the second 40 amino acids. MTS charge was only associated with the acidic residues in the first 40 amino acids of *H. sapiens* and *M. musculus*; there was no association between either region in *S. cerevisiae*, and only the second 40 amino acids showed a correlation in both plant species. This analysis was expanded to determine if the predicted secondary structure in the N-terminal of the mature sequence was different between mature sequences with short or long MTSs (Figure 4; Figure S2). Experimental data does suggest that mature protein unfolding rate is inversely correlated with the stability of the N-terminal of the mature sequence [46] and that protein unfolding rate can be influenced by the secondary structure of N-terminal sequence immediately following the MTS [44]. Two observations were made: short MTSs were more likely to be associated with mature sequences that contain shorter alpha helix and beta strands, whereas mature sequences associated with longer MTS sequences tended to have a greater proportion of alpha helical regions (Figure 4B) and/or an increased occurrence of disordered or unstructured regions (Figure 4D), particularly in the first 50 amino acids of the mature sequence. These characteristics were most prevalent in the *H. sapiens* and *M. musculus* datasets, less prevalent in the *S. cerevisiae* and *O. sativa* datasets, and not obviously present in *A. thaliana*. Although it has been demonstrated in an *in vitro* model that mitochondrial import is more efficient when an alpha helix, rather than a beta strand, follows a targeting sequence in the mature sequence [44], this observation does not seem to be correlated with a change in MTS length and is not consistent across taxa. The prediction that disordered regions, which are characterised by a lack of bulky hydrophobic residues and rich in polar and charged residues, are associated with longer MTSs is somewhat counterintuitive, since such disordered regions are thought to be often unstructured [47] and therefore should be easier to import as they are likely loosely- or un-folded. These regions may however reflect more complex (and therefore difficult to predict bioinformatically) structural configurations that may be difficult to import.

**Table 1:** Correlation analysis between MTS property and N-terminal mature sequence (MTPT) acidic residues relative to the cleavage site.

	MTS length vs MTPT acidic residues		MTS charge vs MTPT acidic residues	
Species	1–40 aa	41–80 aa	1–40 aa	41–80 aa
*H. sapiens*	0.360 (0.001)*	0.152 (0.166)	0.333 (0.002)	0.111 (0.311)
*M. musculus*	0.274 (0.012)	0.109 (0.323)	0.367 (0.001)	−0.004 (0.969)
*S. cerevisiae*	0.349 (0.014)	0.282 (0.05)	0.181 (0.213)	0.185 (0.203)
*A. thaliana*	0.064 (0.716)	0.423 (0.011)	0.102 (0.558)	0.494 (0.003)
*O. sativa*	0.372 (0.025)	0.208 (0.223)	0.029 (0.866)	0.443 (0.007)

* Data represented as Spearman's rho (non parametric) correlation coefficient with two-tailed p-values in parentheses.

Although the statistical mining of primary sequence data can only at best provide indirect evidence of a structure-function relationship, we believe that these observations suggest that negative charge interactions and structural features, particularly in the immediate N-terminal of the mature protein, reflect evolutionary constraints that likely influence the susceptibility or resistance of a protein to be imported. These limitations are consistent with a number of experimental observations, including that the mature portion of mitochondrial protein must be essentially unfolded to allow import [48], that some proteins require active unfolding to ensure they are import competent [49], and that longer MTSs unfold mature proteins faster than shorter variants [50]. It is therefore likely that a proportion of MTS diversity exists to mitigate these constraints in the N-terminal of the mature sequence and overcome potential resistance to unfolding prior to import.

## Conclusions

In this investigation, we present evidence that suggests MTS diversity is influenced in part by physiochemical and structural characteristics of the mature protein that it imports, and that some of these correlated characteristics are evolutionarily maintained across a number of taxa. MTS diversity therefore likely reflects adaptation that balances the engagement of import-promoting mechanisms (such as the electrophoretic effect driven by the inner mitochondrial membrane potential and the matrix import motor, mtHsp70) and the likely import rate-limiting properties of the mature sequence (such as physicochemical interactions between the precursor protein and the import complex, and unfolding potential of the mature sequence prior to import). Charge characteristics of the MTS and mature sequence seem to be most influenced by these adaptations; however, they do not seem to influence plant MTS diversity to the extent seen in non-plant species, which may be due to comparatively longer MTSs in plants that allow greater interaction with mtHsp70 and in turn, possibly less dependency on electrophoretic effect across the inner membrane. Despite the significance of the data presented, a large proportion of the variation among these sequences remains unexplained. This is not necessarily surprising, given that predominantly primary amino acid sequence properties were investigated here. Further understanding of the folding variation among mature sequences, particularly in the N-terminus, will likely account for additional variation among MTS sequences.

## Supporting Information

Figure S1 **Pairwise factor analysis of multivariate PCA.** Blue – *H. sapiens*, Green – *M. musculus*, Biege – *S. cerevisiae*, Purple – *A. thaliana*, Yellow – *O. sativa*.
(EPS)

Figure S2 **N-terminal secondary structure analysis of all mature sequences (excluding** ***H. sapiens*** **) associated the five shortest and five longest MTSs.** See Figure 4 for description.
(PDF)

Table S1 **Quantitative comparison of sequence characteristics and amino acid groupings of MTS and mature protein sequences for each species used in this investigation.**
(PDF)

Dataset S1 **Mitochondrial matrix protein dataset.** A complete list of mitochondrial matrix proteins, gene IDs, accession numbers and both MTS and mature sequences used in this study.
(TXT)

Dataset S2 **Data used to generate Spearman's rho correlation matrix in** **Figure 2** **.**
(TXT)

File S1 **Amino acid frequency analyser (** ***AAResidueFreqCalculator*** **).** Perl script that enables the calculation of both individual and grouped amino acid frequencies from a file containing multiple FASTA sequences. Open the text file for instructions on how to use the program. Convert attached text document file into Perl recognised file by changing the file extension from.txt to.pl (note: requires Perl installed on your system).
(TXT)

File S2 **Reduced amino acid sequence converter (** ***ReducedAASequenceConverter*** **).** Perl script that enables the conversion of amino acidic primary sequence into reduced amino acid sequence. Program will convert multiple FASTA sequences for a text file into reduced alphabet FASTA sequences in a new text file. Open the text file for instructions on how to use the program. Convert attached text document file into Perl recognised file by changing the file extension from.txt to.pl (note: you must have Perl installed on your system).
(TXT)
