# Supplementary material for: Evidence of Evolutionary Constraints That Influences the Sequence Composition and Diversity of Mitochondrial Matrix Targeting Signals
Source: PLoS One. 2013 Jun 25;8(6):e67938. doi: 10.1371/journal.pone.0067938 (PMC3692466; doi:10.1371/journal.pone.0067938)

Figure S2

Species	Accession	MTS Length	Secondary structure of mature protein (1-100 aa) (blue – alpha helix, yellow – beta strand, red – disorder prediction)
Os	Os08g32850.1	18	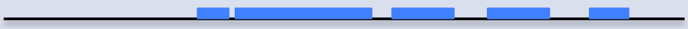
	Os01g46070.1	19	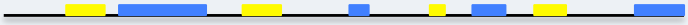
	Os07g09060.1	22	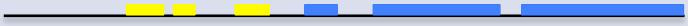
	Os05g03480.2	23	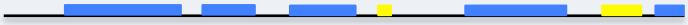
	Os11g01154.1	24	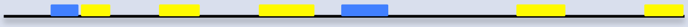
	Os04g32330.1	66	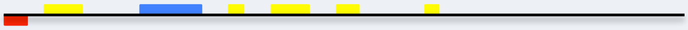
	Os04g44400.1	83	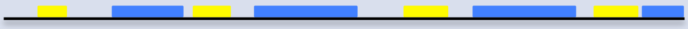
	Os12g32986.1	92	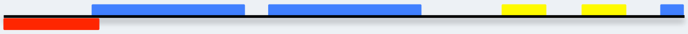
	Os12g41500.1	92	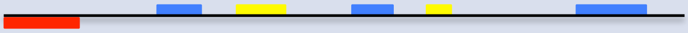
	Os06g01630.1	117	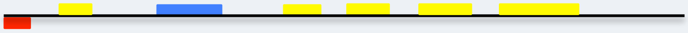
At	AT1G53240.1	22	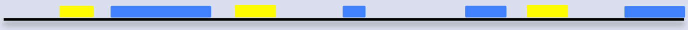
	AT3G61530.1	23	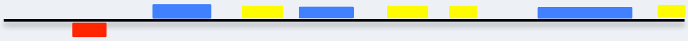
	AT1G59900.1	24	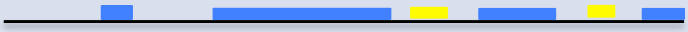
	AT2G35120.1	24	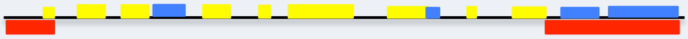
	AT3G10920.1	27	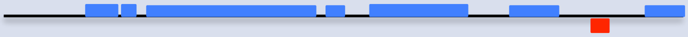
	AT1G51390.1	90	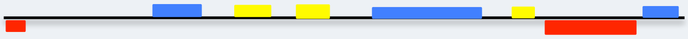
	AT2G05710.1	90	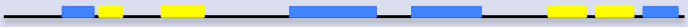
	AT5G52520.1	101	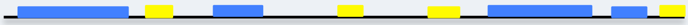
	AT3G13930.1	102	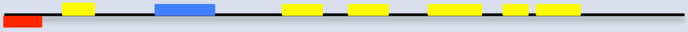
	AT3G59760.1	109	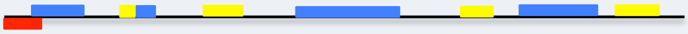

Species	Accession	MTS Length	Secondary structure of mature protein (1-100 aa) (blue – alpha helix, yellow – beta strand, red – disorder prediction)
Sc	ARGJ_YEAST	8	
	ETR1_YEAST	9	
	SYLM_YEAST	9	
	GLO4_YEAST	10	
	JAC1_YEAST	10	
	HEM1_YEAST	56	
	ARG56_YEAST	57	
	CCPR_YEAST	67	
	ODO2_YEAST	71	
	RPM2_YEAST	122	
Mm	HCDH_MOUSE	12	
	SYNM_MOUSE	14	
	PREP_MOUSE	15	
	TMLH_MOUSE	15	
	SYRM_MOUSE	16	
	HEM1_MOUSE	56	
	ODB2_MOUSE	61	
	ADX_MOUSE	64	
	LONM_MOUSE	65	
	C1QBP_MOUSE	70	

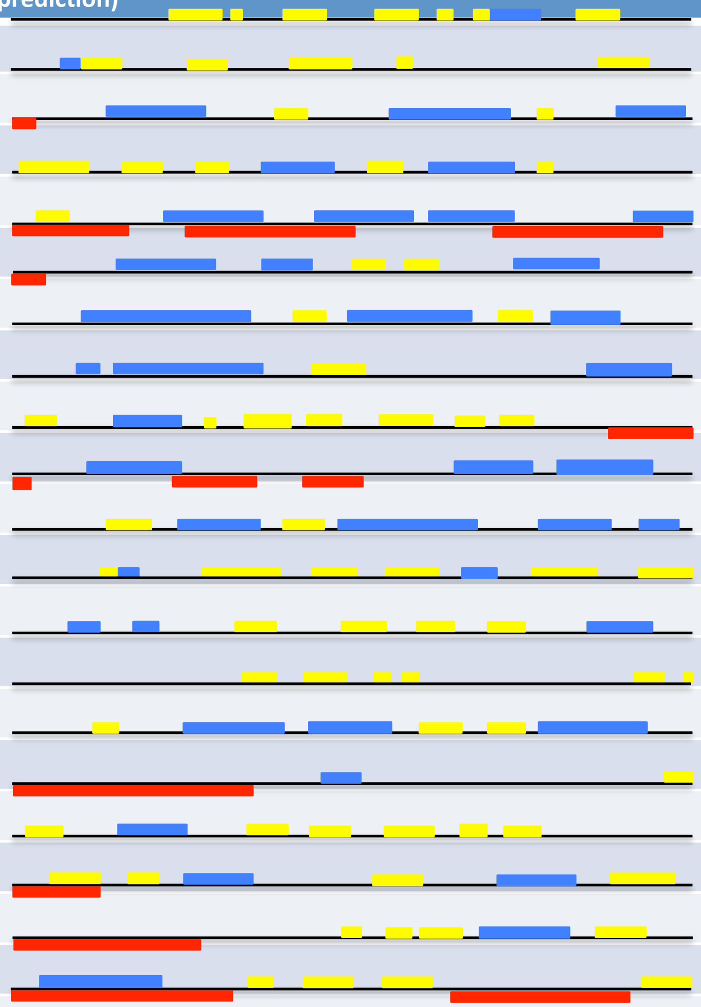

Supplement: Figure S2 — N-terminal secondary structure analysis of all mature sequences (excluding H. sapiens ) associated the five shortest and five longest MTSs. See Figure 4 for description. (PDF) [file pone.0067938.s002.pdf]
