# Supplementary material for: Evidence of Evolutionary Constraints That Influences the Sequence Composition and Diversity of Mitochondrial Matrix Targeting Signals
Source: PLoS One. 2013 Jun 25;8(6):e67938. doi: 10.1371/journal.pone.0067938 (PMC3692466; doi:10.1371/journal.pone.0067938)
Supplement: Table S1 — Quantitative comparison of sequence characteristics and amino acid groupings of MTS and mature protein sequences for each species used in this investigation. (PDF) [file pone.0067938.s003.pdf]

Table S1: Quantitative comparison of sequence characteristics and amino acid groupings of MTS and mature protein sequences for each species used in this investigation

MTS	N	Length	GAVIL	FW	P	CM	ST	Y	NQ	DE	RHK	pH7.5	
H. sapiens	85	34.51 ± 13.66	43.35 ± 10.87	5.50 ± 4.51	6.45 ± 5.09	7.12 ± 3.76	11.72 ± 6.74	1.22 ± 1.85	4.47 ± 4.75	1.38 ± 2.63	18.79 ± 5.02	5.12 ± 1.88	
M. musculus	84	33.08 ± 12.68	41.66 ± 9.84	5.67 ± 4.33	5.28 ± 4.43	7.83 ± 4.08	13.71 ± 6.55	0.89 ± 1.68	4.34 ± 4.40	1.34 ± 2.11	19.29 ± 4.76	4.97 ± 1.62	
S. cerevisiae	56	28.77 ± 19.01	31.69 ± 9.97	6.2 ± 4.36	3.05 ± 3.42	6.91 ± 4.24	19.73 ± 8.5	2.87 ± 3.7	7.63 ± 5.83	0.71 ± 2.42	21.21 ± 5.75	4.94 ± 2.5	
A. thaliana	35	53.6 ± 28.29	34.31 ± 6.67	6.24 ± 3.92	3.81 ± 2.90	6.22 ± 3.33	22.73 ± 6.2	1.03 ± 1.28	5.85 ± 3.25	2.59 ± 3.04	17.23 ± 4.26	5.96 ± 2.45	
O. sativa	36	46.08 ± 28.65	45.5 ± 11.02	2.82 ± 3.01	7.43 ± 6.47	4.49 ± 2.21	12.95 ± 5.29	1.91 ± 2.47	3.50 ± 3.29	2.27 ± 2.84	19.12 ± 5.73	5.87 ± 2.79	
Significant variation?		yes	yes	no	no	no	yes	no	no	no	no	no	
**Mature Protein**	**N**	**Length**	**GAVIL**	**FW**	**P**	**CM**	**ST**	**Y**	**NQ**	**DE**	**RHK**	**pH7.5**	**pI**
H. sapiens	85	399.13 ± 192.79	38.00 ± 4.46	4.99 ± 1.6	4.98 ± 1.37	4.00 ± 1.29	11.31 ± 2.17	2.91 ± 1.09	7.73 ± 1.67	12.25 ± 2.66	13.84 ± 2.22	-1.90 ± 7.16	6.86 ± 1.39
M. musculus	84	481.4 ± 224	37.67 ± 3.4	4.99 ± 1.33	5.07 ± 1.43	4.11 ± 1.05	11.66 ± 1.76	2.96 ± 0.99	7.52 ± 1.4	12.08 ± 2.03	13.95 ± 1.8	-2.78 ± 7.07	6.85 ± 1.16
S. cerevisiae	56	454 ± 251.46	35.62 ± 4.73	4.81 ± 1.45	4.46 ± 1.17	2.89 ± 1.26	12.98 ± 2.09	3.09 ± 1.17	8.7 ± 2.36	13.33 ± 2.86	14.11 ± 2.74	-4.21 ± 9.36	6.71 ± 1.51
A. thaliana	35	398.97 ± 204.93	39.67 ± 3.73	4.09 ± 1.54	4.92 ± 1.72	3.67 ± 1.32	12.9 ± 1.71	2.87 ± 0.99	6.61 ± 1.69	12.55 ± 2.04	12.71 ± 2.07	-6.79 ± 6.12	5.93 ± 0.78
O. sativa	36	427.19 ± 201.14	40.59 ± 4.09	4.3 ± 1.18	5.01 ± 1.71	3.88 ± 1.39	11.89 ± 2.42	2.75 ± 0.93	6.54 ± 1.29	12.22 ± 1.85	12.82 ± 1.55	-7.14 ± 7.25	6.06 ± 0.93
Significant variation?		yes	no	no	no	no	no	no	no	no	no	no	no

[1] Data is presented and the mean ± standard deviation

[2] Significant variation was determined using a two-way ANOVA
